# Exploring the Landscape of Integrative Medicine in Pediatric Oncology: Characterization of an Outpatient Consultative Service

**DOI:** 10.3390/children12020198

**Published:** 2025-02-07

**Authors:** Han-Wei V. Wu, Diana Dominguez Garcia, Julia L. Glade Bender, Jun J. Mao, Nirupa J. Raghunathan

**Affiliations:** 1 Department of Pediatrics, Memorial Sloan Kettering Cancer Center, New York, NY 10065, USA; 2 Albert Einstein College of Medicine, Bronx, NY 10461, USA; 3 Integrative Medicine Service, Department of Medicine, Memorial Sloan Kettering Cancer Center, New York, NY 10065, USA

**Keywords:** children, adolescents, cancer, integrative medicine, complementary therapies

## Abstract

**Background/Objectives**: Symptoms from cancer and treatments often cause pediatric patients and their families to seek complementary and integrative medicine (IM) for relief. The aim of this study was to better describe the characteristics of pediatric patients at a tertiary cancer center who utilize an IM consultative service in the outpatient setting and the associated discussions with a pediatric-focused IM physician. **Methods**: A retrospective study was conducted on initial IM visits for patients aged less than 19 years old at the time of the visit from January 2019 through April 2022 at a tertiary cancer center. Patient demographics, clinical characteristics, and visit information were abstracted from electronic medical records, and discussions with the provider (presenting symptoms and recommendations) were described. **Results**: In total, 207 patients and their associated visit discussions met the criteria. About half (47%, n = 97) of the patients were female with a mean patient age of 10 years old (median age 11 years, range 0 to 18 years). The overall most common presenting symptoms were nausea (35%, n = 72), pain (30%, n = 62), and poor appetite (26%, n = 53) with variations between age groups. The most discussed topics were supplements (94%), diet (91%), stress management (82%), IM therapies (60%), and medical cannabis (54%). **Conclusions**: Priority symptoms reported by patients and referred to the IM outpatient consultative service included nausea, pain, and poor appetite. Concerns were addressed during tailored discussions with patients and their families. Having an outpatient consultative IM service may benefit providers, patients, and families to facilitate receiving evidence-informed recommendations in a dedicated, consolidated setting.

## 1. Introduction

Pediatric patients with cancer frequently experience pain, anxiety, fatigue, and insomnia, which often persist long after treatment [1,2,3]. Patients with cancer and their families increasingly seek complementary and alternative medicine for symptomatic relief, recommendations for healthy lifestyles, immune system boosters, and potential cures [4,5,6]. Between 31% and 84% of pediatric oncology patients worldwide use complementary medicine, including dietary supplements, yoga, and tai chi [7,8].

Therapies that integrate safe and effective non-traditional medical practices with conventional medicine can effectively manage the various side effects experienced by patients with cancer from their treatment regimens [9]. Mind–body techniques can help reduce symptoms of fatigue, pain, stress, and anxiety [10,11,12]. Creative art therapies, such as music and dance/movement, have been shown to improve symptoms of anxiety, stress, and pain [10,11,13]. Massages can also reduce symptoms of pain and associated symptoms, such as insomnia, fatigue, and pain-related interference [14,15,16]. Previous studies have described the utilization of the above-mentioned integrative medicine (IM) therapies in hospitalized children [17,18,19]. However, there are limited studies focused on pediatric patients with cancer who seek out IM services in the outpatient setting.

As defined by an international consensus, “Pediatric Integrative Oncology provides a relationship-centered, evidence-informed personalized approach to the whole child and family system utilizing mind-body practices, natural products, and/or lifestyle modifications alongside conventional oncology care [20]”. Throughout the illness trajectory, Pediatric Integrative Oncology is offered to “optimize health and wellness, enhance healing, minimize suffering, improve quality of life, and empower children and families to become active participants before, during, and beyond cancer treatment [20]”. These holistic aspects of care have increased interest in IM modalities for symptomatic and psychic relief, with cancer centers offering more direct information and incorporated services to patients [21,22].

With the growing demand for IM services at leading centers within the United States [23,24,25], further understanding is required of the types of patients seeking outpatient consultation, their presenting symptoms, and articulated needs. This study seeks to better describe the characteristics of pediatric patients at a tertiary cancer center utilizing an IM consultative service and the content of the associated discussion with a pediatric-focused IM physician.

## 2. Methods

### 2.1. Clinical Population

The IM service at Memorial Sloan Kettering Cancer Center (MSK) established the pediatric consultative service in January 2019 staffed by a pediatric IM physician (N.J.R.). The clinic sees pediatric and adolescent patients, including active cancer patients, survivors, or patients with predisposition syndromes who may be preparing for, undergoing, or have completed treatment. These patients are often referred to the IM outpatient consultative service by a member of their oncology-related primary care team, which can be their primary oncologist, bone marrow transplant physician, and/or surgeon. The clinic visit includes symptom evaluation and management, education on IM therapies and their foundation, recommendations, and referrals to multidisciplinary services, if appropriate. Families and patients may ask questions to the IM physician regarding their primary reasons and symptoms for their referral and solicit advice on potential treatment options for symptom management. Discussion topics may include, but are not limited to, supplements, diet, stress, exercise, or sleep, as well as medical cannabis counseling, research study availability, and IM therapy referral, such as acupuncture, dance/movement, music, mind–body techniques, and touch/massage. Based on patients’ medical and symptom histories, the IM physician will then formulate tailored recommendations, engage the families and patients in a discussion to decide on a final plan via a patient- and family-centered approach, and provide the appropriate education/guidance. After the initial IM clinic visit, the patient and family will decide if they wish to follow through with the recommendations provided and/or IM therapies. Depending on the IM therapy recommended, additional trained specialists may be involved in the patient’s care (i.e., massage therapist, music therapist, etc.). If the patients and families decide to proceed with IM therapies, then IM follow-up visits are coordinated to monitor the treatment process.

### 2.2. Data Abstraction and Analysis

We conducted a retrospective chart review of all initial patient visits to the Pediatric IM clinic from inception through 15 April 2022 (2.3 years). The MSK institutional database (Dataline) was queried and utilized to abstract unique patient information such as demographics and diagnosis. Data were verified through the electronic medical record, utilizing the IM physician notes from initial visits.

Visit data, such as presenting symptoms, active cancer/treatment, and plan recommendations, were manually abstracted from the electronic medical record and inputted into a REDCap (Research Electronic Data Capture, version 14.0.44, hosted at Vanderbilt University) database by the authors. Cancer was denoted as not applicable for patients with predisposition syndromes and diseases in which there was not a primary cancer diagnosis, such as immunodeficiencies or other hematologic conditions. Cancer diagnoses were categorized into three types (solid, liquid, and other) in addition to categorization according to the National Cancer Institute’s Surveillance, Epidemiology, and End Results (SEER) categories of childhood cancers. Treatment was considered active if the patient was within a month of surgery, undergoing or within two weeks of completing radiation therapy, undergoing or within a month of completing chemotherapy, or preparing for or within 2–3 months of receiving a bone marrow transplant. The maximum number of presenting symptoms entered into REDCap was three, with a focus on choosing the most severe and concerning symptom to the patient if more than three symptoms were present.

The verified Dataline information and REDCap database were merged into Microsoft Excel (Version 2308, Build 16.0.16731.20948, Microsoft Corporation, Redmond, WA, USA) and used to gather and report descriptive statistics. Our analysis was limited to patients less than 19 years old at the time of the initial visit to focus on the pediatric and adolescent population. Descriptive statistics were stratified into three different age groups (0–4 years, 5–12 years, and 13–18 years) to account for age-specific differences. This retrospective study was approved by the MSK IRB (17-481).

## 3. Results

The study population consisted of 207 patients with a mean age of 10 years (median age 11 years, range 0–18 years) of whom 47% (n = 97) were female (Table 1). Nearly half of the participants were 13 and older (43.5%, n = 90), the second largest group was 5–12-year-old participants (39.6%, n = 82), and children under 5 accounted for 16.9% (n = 35). Additionally, 83% (n = 172) of patients had a cancer diagnosis and 87% (n = 180) were undergoing active treatment. Most of the cancer diagnoses were solid tumors (76%, n = 157), 16% (n = 34) were liquid cancers, and the remaining 8% (n = 16) were non-malignant diagnoses, such as immunodeficiencies and other hematologic conditions. The type of primary diagnoses varied across age groups. Patients were predominantly White (68%), and the majority were non-Hispanic (81%).

The most common presenting symptoms of the population, as a whole, were nausea (35%, n = 72), pain (30%, n = 62), and poor appetite (26%, n = 53), followed by anxiety, irritability, and fatigue. Symptom profiles varied across age groups (Table 2). Irritability was more common for younger patients, particularly for ages 0 to 4 (40%, n = 14), compared to anxiety and pain, which were reported more among the older-age groups. Gastrointestinal symptoms were commonly reported, with nausea as the top complaint for adolescents (47.8%, n = 43) and poor appetite more common among the younger age groups less than 13 years (29%, n = 34).

**Figure 1:**
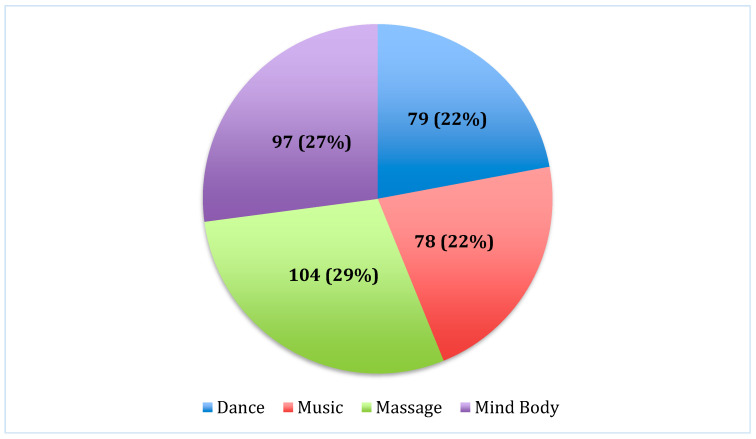
Integrative medicine therapy referral types.

The initial visit discussions included supplements (94%), diet (91%), stress management (82%), IM therapies (60%), and conversations about medical cannabis (54%), as well as exercise and acupuncture (Table 3). IM therapies discussed were dance, music, massage, and mind–body techniques (Figure 1). Supplement discussions included ginger, vitamin D, probiotics, and honey. Discussions around supplements and diet were high across all age groups. Stress (87%, n = 78) and exercise (47%, n = 42) were more frequently discussed in the age group of 13 to 18 years old. IM therapy referrals were more frequently discussed in the age group of 0 to 4 years old (69%, n = 24).

## 4. Discussion

This study provides deeper insights into how Integrative Oncology is utilized in pediatric populations, with a better understanding of presenting symptoms and integrative recommendations. The most common symptoms included nausea (35%), pain (30%), and poor appetite (26%), with varying distributions by age, such as irritability as a top concern in patients under 5 years (40%). The vast majority of patients and/or their parents discussed supplements, diet, and stress management with the integrative medicine physician. Discussions also often included IM modalities such as massage and music therapy, suggesting an interest and inclination for children and their parents to seek therapies outside of prescribed medications to alleviate treatment-related symptoms, even for young age groups.

Consistent with previous studies that described the symptoms that prompted patients with cancer to seek integrative medicine services [26,27,28], our study also found that nausea, pain, and poor appetite were the most reported symptoms. What is novel about this study is its breadth, allowing for the capture of symptoms in patients aged 0 to 4 years for which they were referred to or sought integrative medicine services. For this young age group, irritability was the most common symptom, as opposed to pain in patients aged 5 to 12 years or nausea in 13 to 18 years. Although not widely reported in prior integrative medicine studies, this suggests a potential unique symptom burden and concern in younger pediatric cancer patients who may benefit from the adjunctive support and guidance of a pediatric IM-trained practitioner.

Our study also demonstrates the variety of important discussions and information provided to pediatric patients with cancer and their families by an IM outpatient consultative service. Previous studies have reported that up to 84% of pediatric patients reported using integrative medicine strategies during active treatment [28] and that more than 50% of these patients reported using these therapies up to 5 years post-treatment [29]. While guidelines are set forth by the American Society of Clinical Oncology (ASCO), incorporating the use of integrative medicine in adult cancer care [30,31], there are no official guidelines dedicated to pediatric patients; this is an identified gap. Reliable, high-quality information regarding integrative medicine therapies for symptom management is sought after by pediatric patients, families, and providers to make informed decisions [32,33,34].

A unique aspect of this study was a better understanding of discussion topics between pediatric patients/families and a pediatric-focused IM physician; the most discussed topics involved supplement use (i.e., ginger, vitamin D, probiotics, and honey), diet optimization during cancer treatment, and stress management. Our findings further emphasize that, despite the accessible information available online, there are questions from patients and families that an IM-trained provider in an outpatient consultative service is well equipped to address that other avenues of information, like social media [35], cannot adequately do. Previous studies have reflected physicians’ favorable attitudes toward the use of integrative medicine and highlighted that most practitioners prefer to have a trained IM individual discuss specific uses due to limited knowledge [32,36]. Our findings further underscore the important role that an IM-trained provider can hold in an interdisciplinary care team in providing guidance and reassurance to questions from patients that an oncologist may not feel comfortable with or have the knowledge to answer, such as if certain supplement use may have interactions with chemotherapies patients are receiving, and can help supervise recommended IM therapy use in a safe environment. This type of care model may positively benefit the patient and the patient’s family via addressing and alleviating their concerns and confusion in one consolidated setting throughout their cancer care journey, rather than having patients pull knowledge from a multitude of potentially inaccurate and unverified sources.

This study has limitations. First, this is a single-institution study, which can limit generalizability. This is a retrospective study that gathered data from initial patient visits, which limits our ability to determine if patients and patient families followed up with the recommendations provided post-discussions. We also did not have the data to determine who made the IM referral (oncologist vs. patient self-referral). Finally, since we abstracted visit information for younger-age patients, we acknowledge that these patients’ symptoms or primary concerns would have been reported by a parent or a caretaker, not by the patients themselves. This limitation, particularly for the 0 to 4 age group, is intrinsic to pediatric medicine given that these patients cannot reliably voice their own symptoms due to their developmental stage. Future studies can further explore irritability as a primary concern in the younger age group and track how specific IM therapies targeted for this concern evolved after recommendations were provided.

Our study’s strengths include capturing discussions with a single pediatric-focused IM physician, which reduces the amount of variation for recommendations. Also, this study was conducted at a tertiary cancer center, which sees a high volume of pediatric cancer patients; thus, a diverse spectrum of patient conditions and cancer/treatment-related symptoms are reflected in our findings. Future work can include collecting more granular data on specific recommendations provided to the patient/patient’s families, analyzing patients’ perceptions, and following through with the provider’s recommendations. Additional work can also measure patient outcomes and symptoms after referrals or completion of recommended therapies.

In conclusion, our study highlights the symptoms and presenting concerns that are referred to IM for guidance and demonstrates the breadth of important discussions and information provided to pediatric patients with cancer and their families by an IM outpatient consultative service. Having an outpatient consultative service may be beneficial for patients and families, as well as oncologists, who are seeking reliable information on symptom management and integrative medicine therapies to receive evidence-based recommendations in a dedicated, consolidated setting. Our work also demonstrates the need for further high-quality intervention trials to serve as a foundation for pediatric-oriented IM practice guidelines.

## Figures and Tables

**Table 1:** Demographics and clinical characteristics of population (n = 207).

Characteristic		Total, n (%)	Age 0–4 Years, n (%)	Age 5–12 Years, n (%)	Age 13–18 Years, n (%)
Total		207	35 (16.9%)	82 (39.6)	90 (43.5)
Sex					
	Male	110 (53.1%)	19 (54.3%)	44 (53.7%)	47 (52.2%)
	Female	97 (46.9%)	16 (45.7%)	38 (46.3%)	43 (47.8%)
Race					
	White	141 (68.1%)	21 (60.0%)	54 (65.9%)	66 (73.3%)
	Black	14 (6.80%)	4 (11.4%)	4 (4.9%)	6 (6.7%)
	Asian	19 (9.20%)	2 (5.7%)	11 (13.4%)	6 (6.7%)
	Other	19 (9.20%)	4 (11.4%)	9 (11.0%)	6 (6.7%)
	Unknown	14 (6.80%)	4 (11.4%)	4 (4.9%)	6 (6.7%)
Ethnicity					
	Non-Hispanic	168 (81.2%)	30 (85.7%)	63 (76.8%)	75 (83.3%)
	Hispanic	28 (13.5%)	3 (8.6%)	15 (18.3%)	10 (11.1%)
	Unknown	11 (5.3%)	2 (5.7%)	4 (4.9%)	5 (5.6%)
Cancer Type					
	Solid	157 (75.8%)	29 (82.9%)	64 (78.0%)	64 (71.1%)
	Liquid	34 (16.4%)	1 (2.9%)	10 (12.2%)	23 (25.6%)
	Other *	16 (7.7%)	5 (14.3%)	8 (9.8%)	3 (3.3%)
Undergoing Active Treatment					
	Yes	180 (87%)	31 (88.6%)	72 (87.8%)	77 (85.6%)
	No	27 (13%)	4 (11.4%)	10 (12.2%)	13 (14.4%)

* Other includes cancer predispositions and other hematologic conditions.

**Table 2:** Most concerning presenting symptoms at initial visit.

Presenting Symptoms	Total, n(%)	Age 0–4 Years, n (%)	Age 5–12 Years, n (%)	Age 13–18 Years, n (%)
Total	207	35	82	90
Nausea	72 (34.8)	7 (20.0)	22 (26.8)	43 (47.8)
Pain	62 (30)	11 (31.4)	23 (28.0)	28 (31.1)
Poor Appetite	53 (25.6)	11 (31.4)	23 (28.0)	19 (21.1)
Anxiety	48 (23.2)	4 (11.4)	20 (24.4)	24 (26.7)
Irritability	41 (19.8)	14 (40.0)	16 (19.5)	11 (12.2)
Fatigue	39 (18.8)	4 (11.4)	10 (12.2)	25 (27.8)
Poor Sleep	25 (12.1)	4 (11.4)	12 (14.6)	9 (10.0)
Constipation	21 (10.1)	6 (17.1)	9 (11.0)	6 (6.7)

**Table 3:** Plan discussions with patients during initial visits (n = 207).

Plan	Total, n (%)	Age 0–4 Years, n (%)	Age 5–12 Years, n (%)	Age 13–18 Years, n (%)
Total	207	35	82	90
Supplements	195 (95.2)	33 (94.3)	77 (93.9)	85 (94.4)
Diet	188 (90.8)	29 (82.9)	73 (89.0)	86 (95.6)
Stress	170 (82.1)	25 (71.4)	67 (81.7)	78 (86.7)
IM Therapy Referrals	125 (60.4)	24 (68.6)	47 (57.3)	54 (60.0)
Cannabis	112 (54.1)	21 (60)	43 (52.4)	48 (53.3)
Exercise	60 (29.0)	3 (8.6)	15 (18.3)	42 (46.7)
Acupuncture	20 (9.7)	0 (0.0)	5 (6.1)	15 (16.7)

## Data Availability

The data presented in this study are available upon request from the corresponding author due to patient privacy guidelines.
